# Investigating Ebola virus pathogenicity using molecular dynamics

**DOI:** 10.1186/s12864-017-3912-2

**Published:** 2017-08-11

**Authors:** Morena Pappalardo, Francesca Collu, James Macpherson, Martin Michaelis, Franca Fraternali, Mark N. Wass

**Affiliations:** 1 School of Biosciences, University of Kent, Kent, UK; 2 Randall Division of Cell and Molecular Biophysics King’s College London, London, UK

**Keywords:** Ebola, Molecular dynamics, Virology, Protein structure, Pathogenicity

## Abstract

**Background:**

Ebolaviruses have been known to cause deadly disease in humans for 40 years and have recently been demonstrated in West Africa to be able to cause large outbreaks. Four *Ebolavirus* species cause severe disease associated with high mortality in humans. Reston viruses are the only Ebolaviruses that do not cause disease in humans. Conserved amino acid changes in the Reston virus protein VP24 compared to VP24 of other Ebolaviruses have been suggested to alter VP24 binding to host cell karyopherins resulting in impaired inhibition of interferon signalling, which may explain the difference in human pathogenicity. Here we used protein structural analysis and molecular dynamics to further elucidate the interaction between VP24 and KPNA5.

**Results:**

As a control experiment, we compared the interaction of wild-type and R137A-mutant (known to affect KPNA5 binding) Ebola virus VP24 with KPNA5. Results confirmed that the R137A mutation weakens direct VP24-KPNA5 binding and enables water molecules to penetrate at the interface. Similarly, Reston virus VP24 displayed a weaker interaction with KPNA5 than Ebola virus VP24, which is likely to reduce the ability of Reston virus VP24 to prevent host cell interferon signalling.

**Conclusion:**

Our results provide novel molecular detail on the interaction of Reston virus VP24 and Ebola virus VP24 with human KPNA5. The results indicate a weaker interaction of Reston virus VP24 with KPNA5 than Ebola virus VP24, which is probably associated with a decreased ability to interfere with the host cell interferon response. Hence, our study provides further evidence that VP24 is a key player in determining Ebolavirus pathogenicity.

**Electronic supplementary material:**

The online version of this article (doi:10.1186/s12864-017-3912-2) contains supplementary material, which is available to authorized users.

## Background

The potential of Ebolaviruses to cause large outbreaks has been highlighted by the recent Ebola virus outbreak in West Africa [1] resulting in 28,657 confirmed cases and 11,325 deaths as of 8th May 2016 (http://www.who.int/csr/disease/ebola/en/). To enable replication, viruses depend on mechanisms to antagonise the host cell interferon response. The Ebolavirus proteins that are known to be crucially involved in the suppression of interferon signalling are VP35 and VP24 [2–6]. VP35 prevents interferon signalling by binding and masking double stranded viral RNA. VP24 impairs the host interferon response by binding to the karyopherins α1 (KPNA1), α5 (KPNA5) and α6 (KPNA6) and the transcription factor STAT1 [3–5]. Karyopherins would normally bind STAT1 and transport it to the nucleus, a key step during interferon signalling. VP24 prevents this transport and the subsequent accumulation of STAT1 in the nucleus [2–6].

**Fig. 1:**
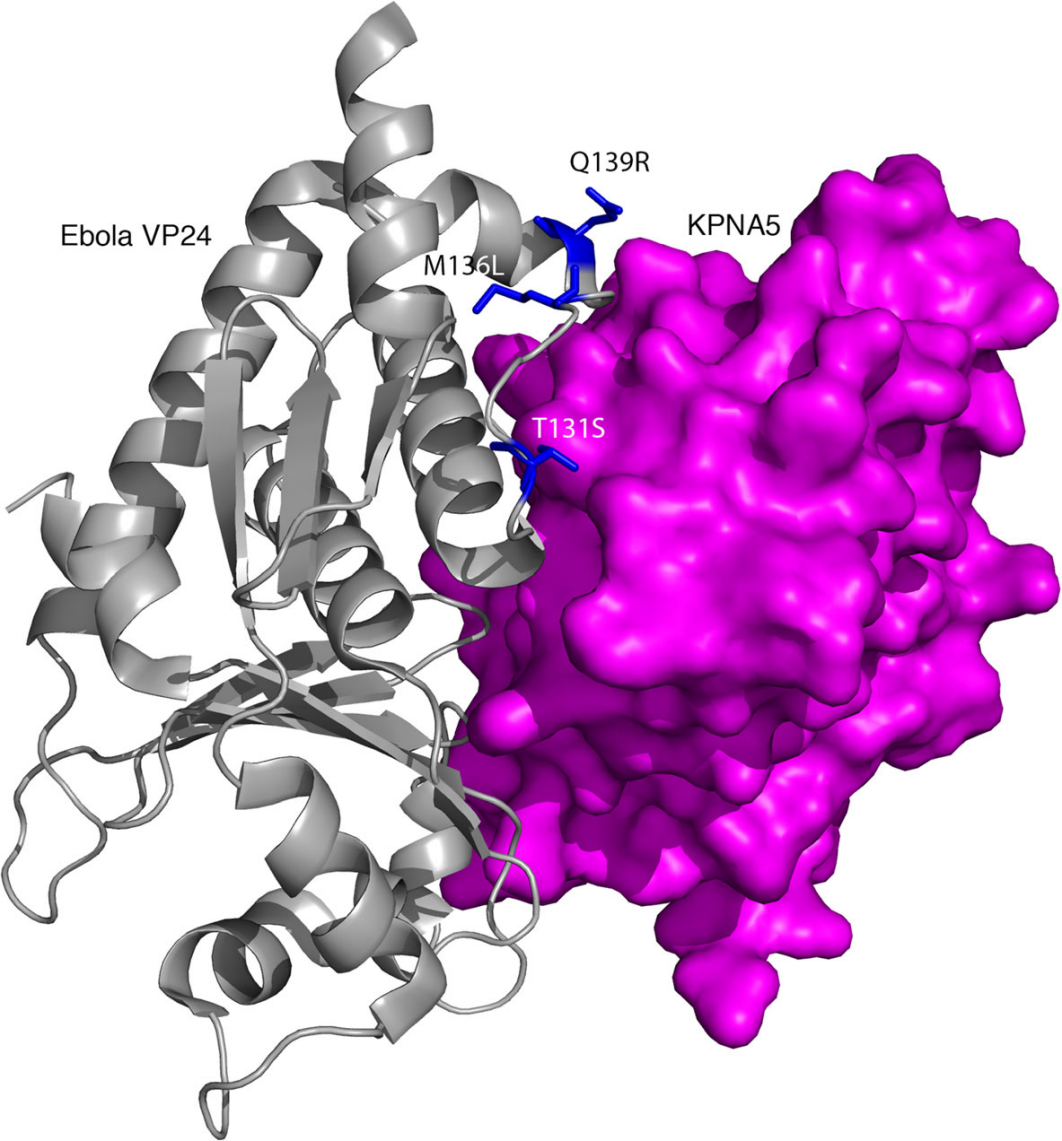
Ebola virus VP24 complex with KPNA5. VP24 is coloured grey and KPNA5 is blue. Residues differentially conserved between Ebola and Reston viruses are shown in red (present in interface), orange (not in the interface site), they are and labelled with the Ebola virus amino acid, residue number followed by the Reston virus amino acid

Of the five known *Ebolavirus* species, only Reston viruses are not pathogenic in humans [7]. A recent study identified genetic variants, that result in amino acid substitutions between the four human-pathogenic *Ebolavirus* species and Reston viruses that may explain the discrepancy in human pathogenicity [8]. Certain amino acid changes (T131S, M136 L, Q139R – Ebola virus residue listed first and Reston virus residue second) in the VP24-karyopherin interface (Fig. 1) were proposed to reduce the affinity of Reston virus VP24 to human karyopherins resulting in a (compared to other Ebolavirus VP24 proteins) reduced ability to inhibit interferon signalling.

In this study, we compared the interaction of Ebola virus and Reston virus VP24 with human KPNA5 using protein structural analysis and molecular dynamics simulations.

## Results

### Analysis of the effect of Ebola VP24 mutations on interaction with KPNA5

The crystal structure of the Ebola virus VP24 complex with KPNA5 is available [3] as well as co-immunoprecipitation studies that have investigated the effect of mutations on the ability of Ebola virus VP24 to bind KPNA5. The experimental data has shown that the Ebola virus VP24 mutations R137A and the combination of R137A,T138A,Q139A strongly reduce VP24-KPNA5 binding. Combined F134A,M136A mutations resulted in an intermediate level of VP24-KPNA5 binding. Other single point mutations (including Q139A that we used as a control) had limited effect on VP24-KPNA5 binding [3].

Initially, we used mCSM [9] and FoldX [10] to predict the effect of the investigated mutations on the stability of the Ebola virus VP24-KPNA5 complex (mCSM, FoldX) and the affinity of the proteins (mCSM) (see methods). mCSM predicted that both point mutations, R137A and Q139A, reduce the binding affinity and the stability of the complex, with the R137A mutation, however, having a greater effect (predicted (ΔΔG −1.07 kcal/mol change in complex affinity) than Q139A (Table 1). Note that for mCSM negative ΔΔG values indicate that the mutation is predicted to destabilise the interaction, while positive values stabilise the interaction. The FoldX predictions agree with mCSM for both point mutations. Additionally, FoldX was able to consider combinations of mutations simultaneously and predicted that both sets of mutations (F134A,M136A, and R137A,T138A,Q139A) reduce stability of the complex with a large reduction of more than −7 kcal/ml for the F134A, M136A combination (Table 1). These predictions are generally in agreement with the experimental observations that R137A and the two sets of multiple mutation reduce binding of Ebola VP24 with KPNA5 and that Q139A results in less pronounced effects [3].

**Table 1:** Predicted effect on Ebola VP24-KPNA5 complex stability by mutations in Ebola VP24

Mutation	mCSM stability (ΔΔG - kcal/mol)	mCSM PP affinity (ΔΔG - kcal/mol)	FoldX stability (ΔΔG - kcal/mol)
R137A	−0.80	−1.07	−0.68
Q139A	−0.39	−0.24	−0.33
F134A,M136A	NA	NA	−7.3
R137A,T138A,Q139A	NA	NA	−1.02

Prediction from mCSM (stability and affinity) and FoldX (stability) were calculated for the mutated complexes

**Fig. 2:**
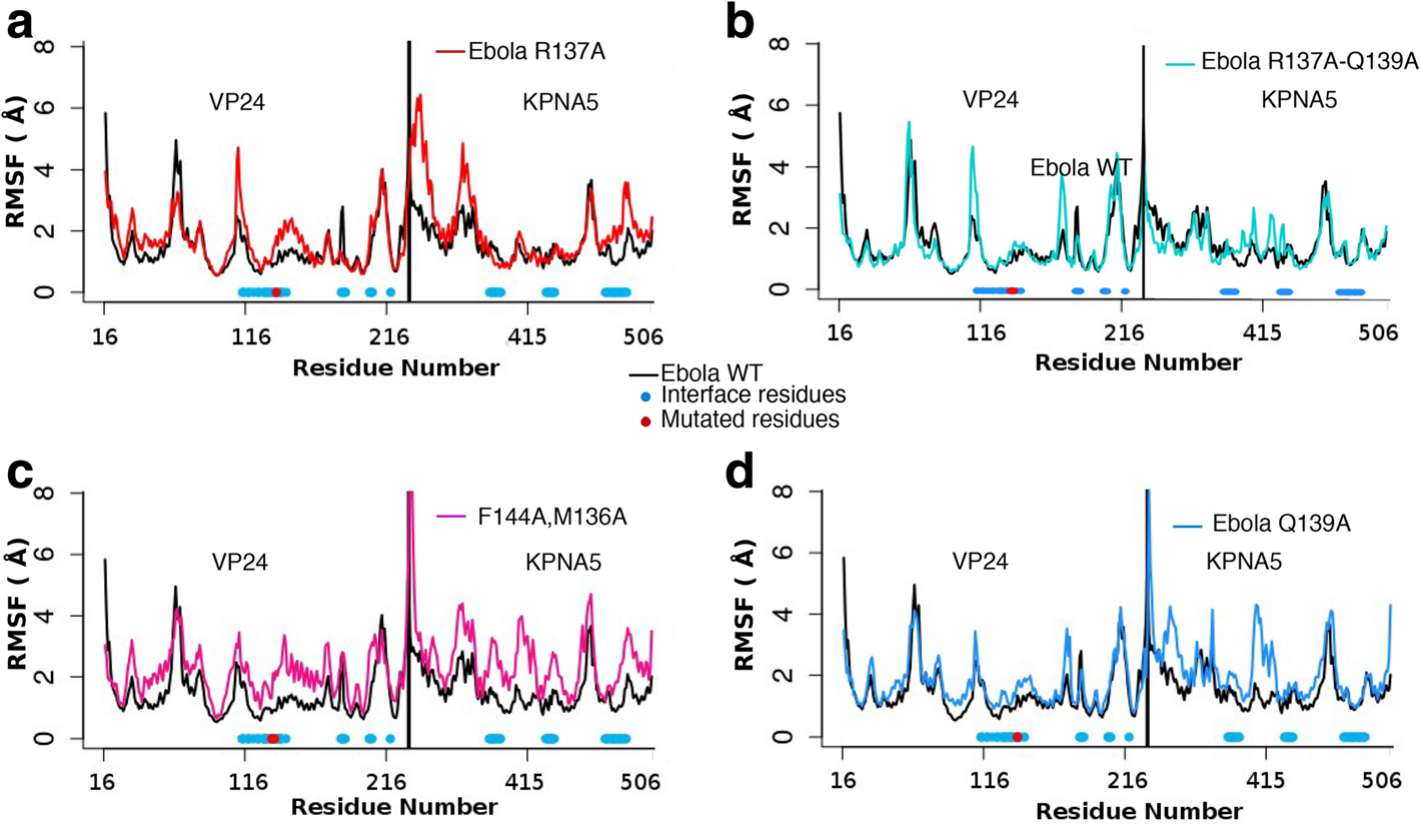
Root Mean Square Fluctuation (RMSF) for simulation of mutated Ebola virus VP24 with human Karyopherin RMSF for the mutants over the wild type complex. EBOV VP24 (on the left side of each graph) and KPNA5 (on the right) are shown. In black line the wild type complex RMSF is shown; in A), the red line shows R137A mutant, in B) the blue line shows the Q139A, in C) the magenta shows F134A-M136A mutant, in D) in cyan R137A-Q139A mutant are shown. In light blue the residues that are occurring at the interface are mapped under each curve

Next, molecular dynamics simulations (MD) for both the wild type Ebola virus VP24 and mutated forms were performed. MD simulations were obtained for the wild type complexes and for the mutated Ebola virus VP24 forms: 1)R137A, 2)Q139A, 3)F134A,M136A and 4)R137A,T138A,Q139A. First the amount of positional fluctuation during the simulation for each residue in VP24 and KPNA5 was considered by calculating the Root Mean Squared Fluctuation (RMSF; Fig. 2). This analysis showed that for all four mutation sets, a greater fluctuation variability is observed in KPNA5. Additionally, for all mutations there is increased fluctuation in VP24 in the interface and in the vicinity of the mutated residues (Fig. 2). This fluctuation is much greater for the three sets of mutations that have an effect on the interaction between VP24 and KPNA5 and smallest for Q139A, which affects binding to a lesser extent (Fig. 2).

### Investigating the effects of VP24 R137A mutation on interaction with KPNA5

According to the experimental data from Xu et al. [3] the single point mutation R137A is the only point mutation that largely removes binding of VP24 to KPNA5. Thus, we used this mutation to validate our system and to obtain additional molecular information on the interaction between the two proteins. The RMSF calculations (Fig. 2) indicated the greatest movement compared to the wild type VP24 centred around residue 115 and also from residues 135 to 150.

**Fig. 3:**
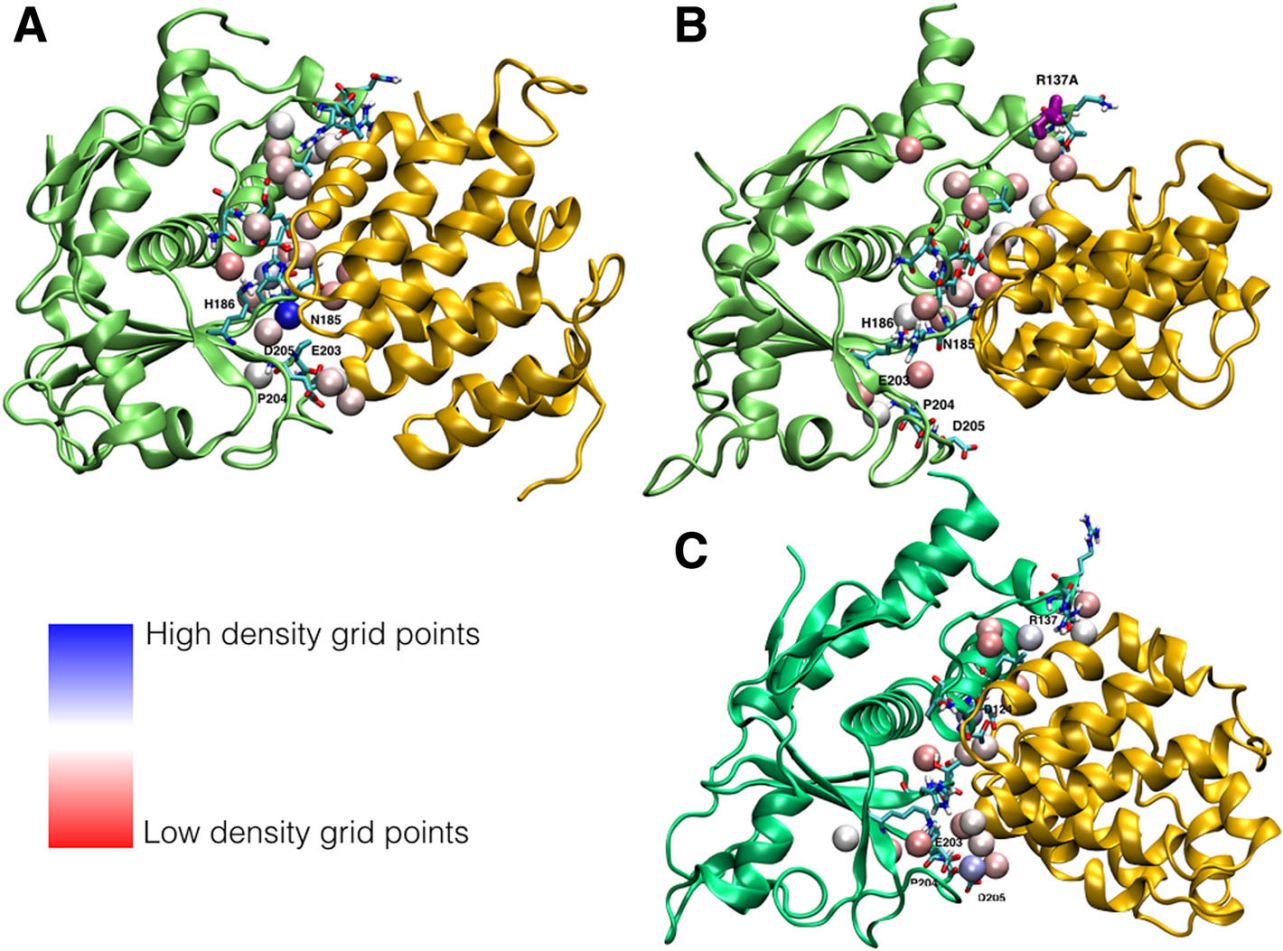
Solvation analysis of the VP24-KPNA5 complexes. In parts **a**, **b** and **c** the spheres represent the most visited grid points coloured from red to blue, with red being a low density grid point (short temporal permanence [ps] of water molecules) and blue a high density grid points (long temporal permanence [ns] of waters). **a**) High density grid points were found close to the residues N185, H186, E203, P204 and D205 in the Ebola virus VP24 with human KPNA5 complex. **b**) The Ebola virus mutant R137A VP24-KPNA5 complex at the interface is characterized only by low density grids points meaning that waters are not trapped and consequently do not contribute to the stabilization of the complex. The mutation induces a long-range destabilization of the complex determining the creation of a cleft at the interface where waters can freely enter and exit. **c**) For the Reston virus VP24 complex, the analysis of solvation at the interface identified high-density grid points close to the residues E203, P204, D205, D124 and R137

To consider how this mutation affects the interaction between VP24 and KPNA5, we analysed the correlation of conformational changes within the complexes and investigated the solvation properties at the interface. The solvation properties for the wild type and R137A-mutated VP24 complexes with human KPNA5 were calculated by estimating the water density on a grid of points constructed around the residues at the interface (see methods). This analysis gave a detailed description of how the solvation influences the binding of VP24 and KPNA5 (Fig. 3).

By considering how frequently each grid point (represented as spheres in Fig. 3) was visited by water molecules, we were able to identify solvation sites at the interface in a continuum range from “low density” grid points (short temporal permanence [ps] of water molecules, red spheres in Fig. 3) to “high density” grid points (long temporal permanence [ns] of water molecules, blue spheres in Fig. 3).

In the wild type Ebola virus complex, high density grid points were found close to the residues N185, H186, E203, P204 and D205 (Fig. 3). This means that at the interface there are solvation sites where water molecules are trapped and contribute to stabilise the complex. In contrast, the mutant R137A VP24-KPNA5 complex at the interface is characterised only by low density grids points, meaning that water molecules do not strongly localise stably at this spot and consequently they are not likely to contribute to the stabilisation of the complex. The mutation induces a long-range destabilisation of the complex creating a cleft at the interface where water molecules can freely enter and exit (Fig. 3b). This is an indication that the fit of the two protomers at the interface of the complex is not optimal. We interpret this conformational change as an early event of the detachment of the two proteins.

The correlation of conformational changes between VP24 and KPNA5 were considered next. Previous studies have found that the binding of a protein ligand to a protein receptor can result in correlated enthalpic backbone motions [11]. Consequently, a model of signal propagation built on the analysis of local motions was generated. These were extracted from the molecular dynamics simulations by encoding trajectories into sequences of 4-residue fragment states with the M32 K25 structural alphabet ([12]; see materials and methods for a full description of the numerical procedure used) to estimate the propensity of the Ebola virus VP24 R137A mutant to remain bound to KPNA5. The local error of the structural alphabet fit for the three complexes is shown in Additional file 1: Figure S3.

**Fig. 4:**
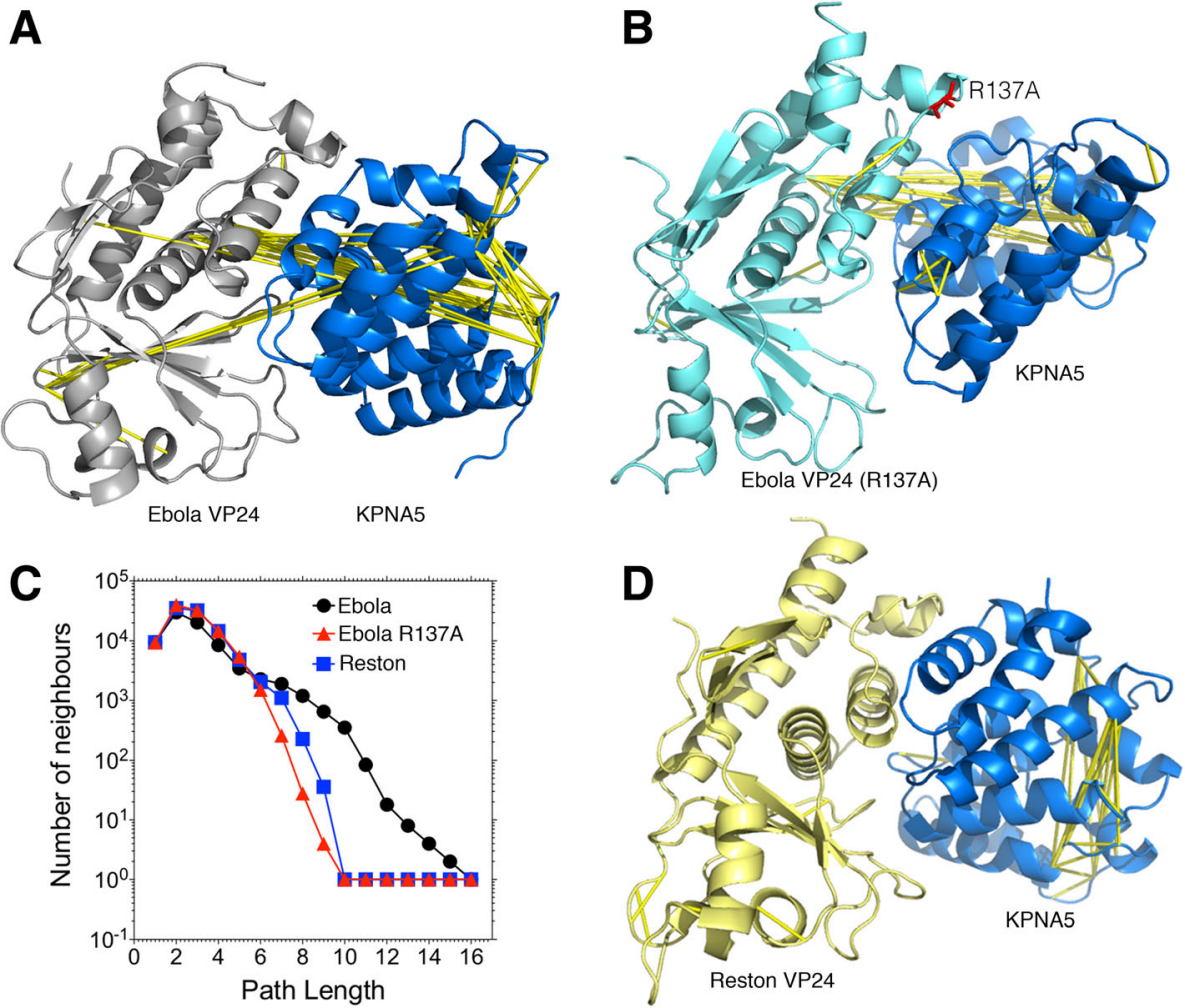
Correlation of conformational Changes in the VP24-KPNA5 molecular dynamics simulations. All-atom molecular dynamic simulations of **a**) Ebola VP24, **b**) Ebola VP24 R137A (shown in *red*) and **c**) Number of inter-subunit correlations at different path lengths **d**) Reston VP24 with human KPNA5 complex were encoded with the M32 K25 structural alphabet and analysed for global correlated motions between distal backbone fragments. The top 10% most significant correlated motions were selected and are represented by yellow strings

The Ebola virus VP24-KPNA5 and Ebola virus VP24(R137A)-KPNA5 complexes were encoded with the M32 K25 structural alphabet and their collective motions were analysed. We assessed the quality of fragment encoding of the simulations by measuring the local fit root-mean square deviation of the fragments from the atomistic trajectories (Fig. 3). We found that the fragment-based encoding produced simplified trajectories with a reasonable special accuracy (>1.0 Å) for each of the trajectories analysed. A statistical analysis of the global collective motions revealed that the Ebola virus VP24-KPNA5 complex displayed structurally correlated motions between distal fragments (Fig. 4a). The molecular dynamics simulation of the mutant VP24(R137A)-KPNA5 interaction displayed fewer such collective motions between the two subunits in the dimer (Fig. 4b). In support of this observation, a topological analysis of the three simulations revealed that the Ebola virus wild type VP24 complex displayed a denser network of inter-subunit correlations than the VP24(R137A) complex (Fig. 4). Taken together, the physical model presented here would suggest that the wild type Ebola virus complex is molecularly optimised for dimerisation, whereas we predict that the mutant VP24(R137A) is unlikely to form a stable complex, which is in agreement with the experimental data from Xu et al. [3].

### Comparison of Ebola virus and Reston virus VP24 interaction with KPNA5

Next, the complex formed between Reston virus VP24 and KPNA5 was investigated with the aim of identifying how the interaction between these two proteins may differ from the interaction between Ebola virus VP24 and KPNA5. Only an unbound structure of Reston VP24 was available. Hence, a model of Reston virus VP24 bound to KPNA5 was generated using the Ebola virus VP24-KPNA5 complex as a template (see Methods).

mCSM predicted that all of the conserved amino acid changes observed in Reston virus VP24 would reduce the stability and affinity of the complex, with the exception of M136 L, where a small increase in affinity was predicted (Table 2). The other changes in stability were similar to the predicted change for R137A, which is known to reduce binding [3]. FoldX also predicted reduced stability for two (T131S, N132 T) of these four point changes, with increased stability predicted for M136 L and Q139R, although the ΔΔG for M136 L is predicted to be very small (0.18 kcal/mol). FoldX also predicted a slightly less stable complex with all four amino acid changes present (Table 2). Overall these predictions suggest that together the amino changes at the Reston virus VP24-KPNA5 interface are likely to reduce the stability and affinity of the complex.

**Table 2:** Predicted effect on Ebola virus VP24-KPNA5 complex stability by mutation of residues that are differentially conserved in Reston in Ebola VP24

Mutation	mCSM stability (ΔΔG - Kcal/mol)	mCSM PP affinity (ΔΔG - Kcal/mol)	FoldX stability (ΔΔG - Kcal/mol)
T131S	−1.29	−0.32	−0.42
N132 T	−0.62	−2.65	−1.22
M136 L	−0.81	0.17	0.18
Q139R	−1.06	−0.99	1.59
T131S,M136 L,Q139R	NA	NA	−0.3

Prediction from mCSM and FoldX are shown changes for the in the EBOV VP24 –KPNA5 complex

**Fig. 5:**
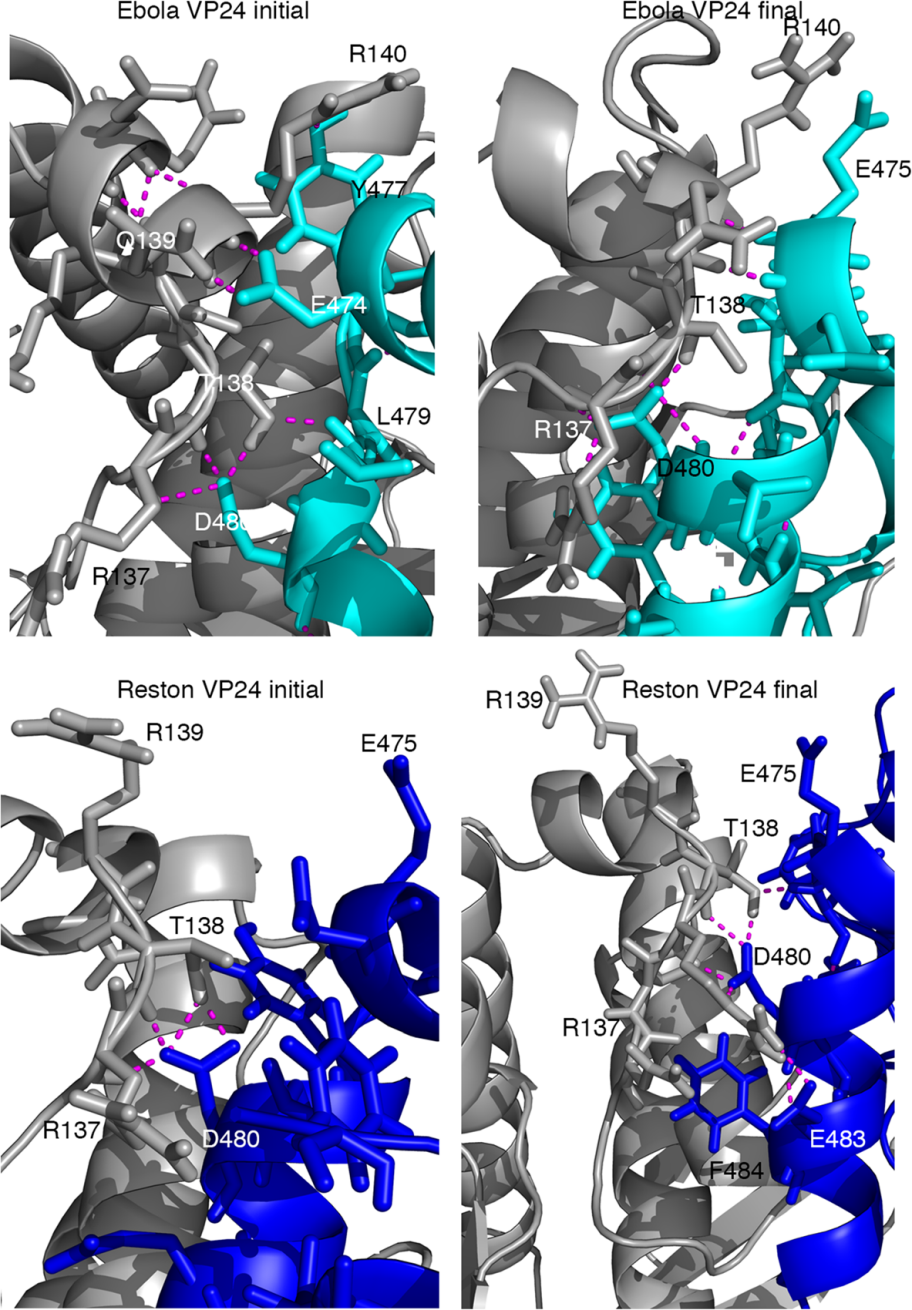
Hydrogen bonding in the Ebola and Reston virus VP24 complexes with KPNA5. VP24 hydrogen bonding with KPNA5 in the region of residues R137-R140. Top two panels are for VP24 with the initial minimised structure (*left*) and the structure at the end of the simulation (*right*). Bottom panels show the equivalent for Reston VP24. In all images VP24 is coloured grey

The interfaces of the Ebola virus VP24- and Reston virus VP24-KPNA5 complexes were compared (Table 3) at the beginning and end of the molecular dynamics simulation using PISA [9] and POPSCOMP [10]. Both complexes are stabilised by patterns of hydrogen bonds. In the X-ray structure of the Ebola virus VP24-KPNA5 complex, nine hydrogen bonds were present, while in the model of the Reston virus VP24-KPNA5 complex 11 hydrogen bonds were identified to form at the interface. In total three hydrogen bonds were equivalent in the two complexes for the energy minimised structures (i.e. at 0 ns). Molecular dynamics simulations for Ebola or Reston virus VP24 in complex with KPNA5 were then performed for 600 ns. We calculated the number of hydrogen bonds at the interface over the whole trajectory (600 ns) for both complexes (Additional file 1: Figure S4). From the probability distribution of the number of hydrogen bonds over the time, we observed that the Ebola virus VP24-KPNA5 complex is stabilised by 22 hydrogen bonds on average compared to 18 for the Reston virus VP24-KPNA5 complex (Additional file 1: Figure S4). So during the simulation the complexes have a greater number of hydrogen bonds than the starting structures. The four fewer hydrogen bonds at the interface of the Reston virus VP24-KPNA5 would suggest that this complex is energetically weaker than the Ebola virus VP24-KPNA5 complex. Analysis of the simulations highlighted that in the Ebola virus VP24 complex there was a considerable hydrogen bonding network with KPNA5 (Fig. 5b) involving VP24 residues 137–140. However, in the Reston virus VP24-KPNA5 complex there was only limited hydrogen bonding of VP24 R137 and T138 with KPNA5 D480 (Fig. 5b). In addition, the Ebola virus VP24-KPNA5 complex contains a hydrogen bond between VP24 Q139 and KPNA5 E474. In contrast, VP24 R139 points away from KPNA5 and towards the solvent in the Reston virus VP24-KPNA5 throughout the simulation (Fig. 5b).

**Table 3:** PISA and POSPCOMP Interface Analysis at 0 and 600 ns MD snapshots

	Ebola VP24–KPNA5 complex	Reston VP24–KPNA5 complex
PISA results		
PISA results at 0 ns		
Interface Area (Å^2^)	1099.7	1055.1
Solvation Free Energy (ΔiG, Kcal/M)	−8.5	−8.6
Hydrogen bonds	9	11
PISA results at 600 ns		
Interface Area (Å^2^)	1119.2	1076
Solvatation Free Energy (ΔiG, Kcal/M)	−10	−9.1
Hydrogen bonds	7	11
POPSCOMP results after minimisation		
Hydrophobic difference (Å^2^)	1042.28	1002.35
Hydrophilic difference (Å^2^)	772.73	713.95
Total difference (Å^2^)	1815.06	1716.43

Throughout the simulation, the RMSD of the main chain C-Alphas was stable for both complexes (Additional file 1: Figure S1). The RMSD of the Reston virus VP24-KPNA5 model is greater than of the Ebola virus VP24-KPNA5 complex, (Additional file 1: Figure S1). This could indicate a difference in the interaction between RESTV VP24 and KPNA5 and could also partly reflect that the simulation is based on a model rather than a solved structure.

For VP24 some minor differences in fluctuation (RMSF) were observed between the Reston virus and Ebola virus proteins. Two of these differences coincided with the interface site at residue 113 (Fig. 2a). Residue 113 is located in an alpha helix at the interface. For KPNA5 there are larger differences in RMSF in four regions, three of which coincide with the complex interface (Fig. 2a). The most pronounced difference is around residues 477–479 (a loop region between two alpha helices), where there is very little fluctuation of KPNA5 in the Ebola virus VP24 complex (around 1 Å) but a peak fluctuation of 8 Å in the Reston virus VP24 complex. The greater fluctuation in KPNA5 suggests that the interaction with Reston virus VP24 differs from that with Ebola virus VP24, supporting the evidence that Reston virus VP24 and human KPNA5 are weaker binding partners than Ebola virus VP24 and human KPNA5.

Analysis of the secondary structure (using DSSP [13]; see methods) during the simulation revealed minor changes in the VP24 secondary structure occurring at the interface site (Additional file 1: Figure S2). The most important changes were found around residue 76 where there is a prevalence of turns in Ebola virus VP24 which is a coiled structure in Reston virus VP24. Residues 133 and 134 (Additional file 1: Figure S2), as well as residue 146, which are proximal to the binding interface, lose their bend and beta bridge structures to become unstructured in the Reston virus complex. The largest changes in secondary structure were found in KPNA5, particularly in two regions between residues 365–375 and 385–395 (Additional file 1: Figure S2). The second region, which is involved in binding VP24, loses alpha helical structure after 220 ns in the Reston virus complex and changes to a turn structure instead.

For the Reston virus VP24-KPNA5 complex, analysis of solvation at the interface identified high-density grid points (visited by permanent water molecules) close to the residues E203, P204, D205, D124 and R137 (Fig. 3c). As in the case of the wild type Ebola virus VP24-KPNA5 complex, at the interface there are solvation sites where water molecules are trapped and contribute to the stabilisation of the complex. In both the wild type Ebola virus and Reston virus VP24-KPNA5 complexes high-density grid points were found close to the residues E203, P204 and D205. This means that these residues are important in enhancing the stability of both complexes while establishing favourable interactions with water molecules. These residues belong to a loop interacting with KPNA5 defining a cavity where the water molecules are trapped.

The presence in the Reston VP24-KPNA5 complex of high-density grid points, where water molecules are trapped, suggests that part of the interface between Reston virus VP24 and KPNA5 may be relatively stable compared to the R137A-mutated Ebola virus VP24-KPNA5 complex, where only low density grid points were observed, indicating an absence of water molecules that would stabilise the complex (Fig. 3).

Correlation of conformational changes in the Reston virus VP24-KPNA5 complex was compared to the results for the wild type and R137A mutant Ebola virus VP24-KPNA5 complexes. No correlated motions were detectable in the simulation of the Reston virus complex variant of the VP24-KPNA5 complex (Fig. 4c), whereas many were observed for the Ebola virus VP24-KPNA5 complex and fewer for the Ebola virus R137A VP24-KPNA5 complex (see above). Correlation of the conformational changes analysis supported this with the Ebola virus VP24-KPNA5 complex having a denser network of inter-subunit correlations than did the Reston virus VP24-KPNA5 complex, which reflects the results obtained for the R137A mutant Ebola virus VP24-KPNA5 complex (Fig. 4). Taken together, these findings suggest that Reston virus VP24 forms a less stable complex with KPNA5 than Ebola virus VP24, in particular due to the similarity of the results obtained for Reston virus VP24 and R137A-mutated Ebola virus VP24, which is known not to bind human KPNA5 [3].

## Discussion

This study investigated the dynamics of interaction of the Ebolavirus protein VP24 with human KPNA5. This interaction occurs during infection to prevent transport of STAT1 to the nucleus in order to inhibit interferon signaling [3–5]. Recent findings have identified positions that are differentially conserved between human-pathogenic Ebolaviruses and Reston virus, the only Ebolavirus that is not pathogenic to humans [8]. In particular three of these positions in the interface site between VP24 and human KPNA5 were suggested to affect binding of Reston virus VP24 to KPNA5 and to contribute to the lack of human pathogenicity of Reston viruses [8].

Experimental evidence showed the R137A mutation in Ebola virus VP24 to reduce VP24 binding to human KPNA5 [3]. Therefore, we compared the Ebola virus VP24-KPNA5 interaction with the R137A-mutant Ebola virus VP24-KPNA5 interaction to validate our system. In concert with the experimental findings [3], our analysis predicted that the R137A mutation reduces VP24-KPNA5 binding. Moreover, the correlations of conformational changes and solvation analysis provide novel molecular insights into how this mutation affects binding of the two proteins. They show that the mutation reduces the stability of the complex, enables the two proteins to move apart from each other, and for water to enter the interface.

The investigation of the interaction of Reston virus VP24 with human KPNA5 added further evidence that Reston VP24 is a weaker binding partner to human KPNA5 than Ebola virus VP24. This is most strongly suggested by the lack of correlated conformational movements between Reston virus VP24 and KPNA5. Some permanent water molecules are (in contrast to the R137A-mutated Ebola virus VP24-KPNA5 complex) observed in the interface of the Ebola virus VP24- and Reston Virus VP24-KPNA5 complexes. This indicates some differences between the interaction of R137A-mutated Ebola virus VP24 and Reston virus VP24 with human KPNA5.

## Conclusion

In conclusion, our results suggest that Reston virus VP24 forms a weaker complex with human KPNA5 than Ebola virus VP24. This weaker binding is anticipated to reduce (in comparison to Ebola virus VP24) the capacity of Reston virus VP24 to inhibit KPNA5-mediated STAT1 transport into the nucleus and to anatagonise interferon signalling in human cells. This reduced Reston virus VP24-KPNA5 complex stability is likely to contribute to the lack of human pathogenicity observed in Reston viruses. Hence, our findings contribute novel evidence indicating VP24 to be an important regulator of species-specific Ebolavirus pathogenicity, as previously suggested by the analysis of conserved differences between Reston viruses and human-pathogenic Ebolaviruses [8]. In addition, our findings provide novel mechanistic insights at the molecular level on the interaction of Ebolavirus VP24 with human KPNA5.

## Methods

### Modelling of a RESTV-VP24 KPNA5 complex

The Ebola and Reston virus VP24 sequences share 81.3% sequence identity and 96% similarity. The protein structures were aligned using Chimera [14] and a model for Reston VP24 in complex with human KPNA5 built using MODELLER 9.0 [15]. The Reston virus VP24 crystal structure (PDB 4D9O) and the Ebola virus VP24-KPNA5 complex (PDB 4U2X) were used as templates for the Reston virus complex model. 200 models were obtained and the one with the lowest DOPE score was selected.

### Comparison of interfaces

PISA [9] and mCSM [9] were used to analyse the structural properties at the complexe interfaces, including solvent accessibility and binding affinity. FoldX [10] was used to predict the effects of the changes in Energy upon mutation, in terms of effects in protein stability. POPSCOMP [16] was used to determine the contribution of the individual residues to the hydrophilicity and hydrophobicity at the interface, according to their solvent accessible surface area (SASA), using default parameters. The residues were classified as being part of the core, support or rim regions of the interface according to the change in SASA (when the percentage of hydrophobicity was greater than 40 and difference in SASA was less than 10 Å^2^ the residue was considered as core, otherwise it was rim).

### Molecular dynamics simulations

Molecular dynamics simulations were performed for the wild type forms of Ebola virus VP24 and Reston virus VP24 in complex with human KPNA5. Other simulations were performed on the Ebola virus VP24-KPNA5 complex with mutations introduced into VP24 where the effect on KPNA5 binding had been experimentally determined [3]. The mutations considered were: 1)R137A, 2)Q139A, 3)F134A,M136A and 4) R137A-Q139A.

Molecular dynamics simulations were performed using Gromacs 5.0.5 [17]. The procedure used has been previously described [16]. Briefly, starting structured were solvated in a cubic box of SPC water and the distance between the protein and box boundaries was set to a minimum of 12 Å. The standard protonation state (pH 7) for ionisable residues was used, with counterions used to neutralise the system. The GROMOS 53a6 parameter set [18] was used. Periodic boundary conditions were imposed. Temperature and pressure regulation was performed using the Berendsen algorithm [19] using coupling constants of 0.2 and 1 ps respectively. The Ewald method [20] (particle mesh) was used to calculate electrostatic interactions. The neighbour list for non-covalent interactions were updated every five steps. The first minimisation used the 1000 steps of steepest descent. Harmonic positional restraints were applied to the Heavy atoms in the protein (initially 4.8 kcal/mol/Å-2, reduced to 1.24.8 kcal/mol/Å-2) and the temperature (at constant volume) increased from 200 K to 300 K. The simulation was then performed for 100 ps at constant temperature and pressure (300 K and 1 bar). System coordinates were saved every 1 ps.

We selected the force field GROMOS 53a6 because is the latest version of the widely used force field [21] from the GROMOS family stemming from the most widely used GROMOS 43A1. These parameter sets are often selected in performance studies where force fields are assessed and compared [22–25], and where it is shown that it is still one of the best united-atom force fields available, providing in some cases even better results than some all-atom force fields. Additionally, it is known since a while that the 43A1 parameter set provides better alpha/beta relative stabilities than GROMOS parameter sets developed later [26].

600 ns MD trajectories were obtained for the Ebola virus VP24-KPNA5 complex and the model of the Reston virus VP24-KPNA5 complex. In our analysis, we omitted the first 280 ns of the simulation, as this was the approximate time required for each of the systems to reach conformational equilibrium. 200 ns trajectories were obtained for R137A and F134A, M136A and 100 ns for all other simulations.

### Molecular dynamics analysis

Trajectories were analysed using the GROMACS analysis tools, VMD tools and the Bio3D package for R [27, 28]. For the analysis, standard Periodic Boundary Conditions were removed and Minimum Image Convention (MIC) were applied to all the trajectories. Rotational and translational movements were then deleted in order to perform the Principal Component Analysis. Secondary structure plots for trajectories were obtained using the DSSP [29] tool in gromacs. Root mean square deviation (RMSD) and fluctuation (RMSF) from the initial starting complex were obtained using Bio3D, as well as the PCA analysis and correlation plots.

### Analysis of correlation of conformational changes

We used a fragment-based approach to simplify the local structure of each of the three molecular dynamics simulations. The structural alphabet M32 K25 was used to encode fragments of four consecutive C^α^ atoms, so as to describe backbone conformations of the protein in a simplified manner [12]. Mutual information between two sets of fragments (*i,j*) was used to calculate the correlation in conformational changes using a numerical procedure outlined in [12]:

$$ {I}^n=\frac{I\left({C}_i;{C}_j\right)-\epsilon \left({C}_i;{C}_j\right)}{H\left({C}_i,{C}_j\right)} $$

where *C*_*i*_ and *C*_*j*_ are columns in the structural string alignment, *I(C*_*i*_ *;C*_*j*_ *)* is the mutual information, *H(C*_*i*_ *;C*_*j*_ *)* is the joint entropy and *ε(C*_*i*_ *;C*_*j*_ *)* is the error.

Considering the fragment at positions *C*_*i*_ and *C*_*j*_ as a discrete distribution of fragments *X* and *Y*, we calculated the joint entropy *H(X,Y)* as a pair of discrete random variables *(X,Y)* with a joint distribution *p(x,y)* defined as

$$ H\left( X; Y\right)=-\sum_{x\varepsilon X}\sum_{y\epsilon Y} p\left( x, y\right) logp\left( x, y\right) $$

where *p(x)* and *p(y)* are the associated marginal probabilities.

### Analysis of solvation in the interaction surface

To perform the water density analysis, structures were simulated, as previously described, for 5 ns by MD with backbone restraints (1.2 kcal· mol − 1· Å − 2) to avoid any significant conformational changes of the protein during the simulation [11, 27]. Water density maps were calculated at discrete points **r** defined by a 0.5-Å spaced rectangular grid around the solute. To remove the overall roto-translational motion of the protein the structures of the last 10 ns of the trajectory were superimposed to a reference. From snapshots taken every 0.1 ps, the density of the water oxygen atoms was averaged for each grid point and normalised by the bulk density evaluated in a 6–8 Å shell around the solute. The hydration score *S*_*hyd*_ was defined by identifying hydration sites as the local maxima of the density map with g(**r**) > 1 as previously described [30]. Water density maps were calculated for representative conformations of the Ebola virus VP24-KPNA5 complex as extracted from the trajectory and compared to the solvent distribution for the representative conformations of Ebola virus R137A VP24-KPNA5 and Reston virus VP24-KPNA5 complex trajectories.

A good water model to use as a solvent in biomolecular simulations should be computationally efficient and at the same time reproduce accurately enough the properties of bulk water. Not to be underestimated, this model should be compatible with the force field used for the solute interactions. It is well known that simple effective pair potentials such as SPC, TIP3P and TIP4P are, in different measure, not able to accurately describe the entire range of water properties, nevertheless, they have all proven to successfully model water as a solvent in biomolecular simulations.

The general weakness demonstrated by these models is in the overestimation of the diffusion coefficient and the inaccurate description of the dielectric properties. On the other hand they are effective in the calculation of solute solvent energies and practical to use. In the past we have developed solvent density maps based on the SPC model [30, 31]. We were particularly interested in the water-protein interactions due to the localization of water at the surface of the protein, therefore we used the same model in this application as a matter of consistency with the force field and our previous calculations.

## Additional file

Additional file 1:
contains Figures. S1-S4. Legends for each of the figures are provided below: **Figure S1.** RMSD curves for VP24 KPNA5 complex trajectories. A) Ebola (black line) and Reston (red line) in complex with KPNA5 are shown. The Reston complex showed a higher RMSD that could be explained with the suboptimal binding between the two monomers (red line), while the EBOV complex showed a lower RMSD during the whole 600 ns trajectory, meaning a greater stability of the complex. B) For Ebola VP24 in complex with KPNA5 for wild type and the set of mutated Ebola VP24 proteins. **Figure S2.** DSSP graphs for the two wild type complexes. On the left side of the graph, the evolution of the EBOV secondary structure is shown in EBOV VP24 (on the top) and KPNA5 (on the bottom). On the right side, the evolution of the RESTV secondary structure is shown for RESTV VP24 (on the top of the graph) and KPNA5 (on the bottom). Residues at the interfaces are mapped in yellow circles. **Figure S3.** Local error of structural alphabet fit. The RMSD distribution per fragment position was calculated for a) WT EBOV VP24 and KPNA5, b) R137A EBOV VP24 and KPNA5 and c) RESTV VP24 and KPNA5. **Figure S4.** Hydrogen bonding in the Ebola and Reston virus VP24 complexes with KPNA5. Probability distribution of the number of H-bonds at the interface during the simulation for Ebola virus VP24- KPNA5 complex (black) and for Reston virus VP24- KPNA5 (red). (PDF 1132 kb)
