# Supplementary material for: Investigating Ebola virus pathogenicity using molecular dynamics
Source: BMC Genomics. 2017 Aug 11;18(Suppl 5):566. doi: 10.1186/s12864-017-3912-2 (PMC5558184; doi:10.1186/s12864-017-3912-2)
Supplement: Additional file 1: — contains Figures. S1-S4. Legends for each of the figures are provided below: Figure S1. RMSD curves for VP24 KPNA5 complex trajectories. A) Ebola (black line) and Reston (red line) in complex with KPNA5 are shown. The Reston complex showed a higher RMSD that could be explained with the suboptimal binding between the two monomers (red line), while the EBOV complex showed a lower RMSD during the whole 600 ns trajectory, meaning a greater stability of the complex. B) For Ebola VP24 in complex with KPNA5 for wild type and the set of mutated Ebola VP24 proteins. Figure S2. DSSP graphs for the two wild type complexes. On the left side of the graph, the evolution of the EBOV secondary structure is shown in EBOV VP24 (on the top) and KPNA5 (on the bottom). On the right side, the evolution of the RESTV secondary structure is shown for RESTV VP24 (on the top of the graph) and KPNA5 (on the bottom). Residues at the interfaces are mapped in yellow circles. Figure S3. Local error of structural alphabet fit. The RMSD distribution per fragment position was calculated for a) WT EBOV VP24 and KPNA5, b) R137A EBOV VP24 and KPNA5 and c) RESTV VP24 and KPNA5. Figure S4. Hydrogen bonding in the Ebola and Reston virus VP24 complexes with KPNA5. Probability distribution of the number of H-bonds at the interface during the simulation for Ebola virus VP24- KPNA5 complex (black) and for Reston virus VP24- KPNA5 (red). (PDF 1132 kb) [file 12864_2017_3912_MOESM1_ESM.pdf]

Investigating Ebola virus pathogenicity using Molecular Dynamics
Supplementary Figures

Morena Pappalardo[1§], Francesca Collu[2§], James Macpherson[2], Martin Michaelis[1], Franca Fraternali[2*], Mark N Wass[1*]

1. School of Biosciences, University of Kent, Kent, UK.
2. Randall Division of Cell and Molecular Biophysics King's College London

A

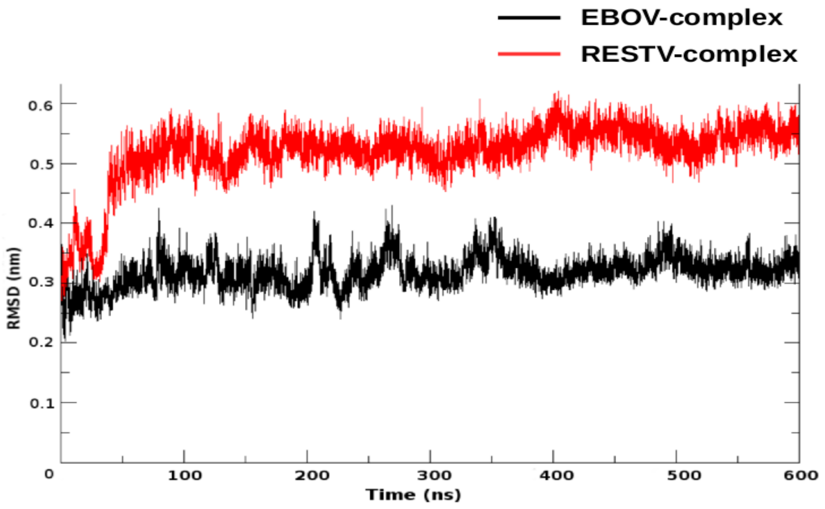

B

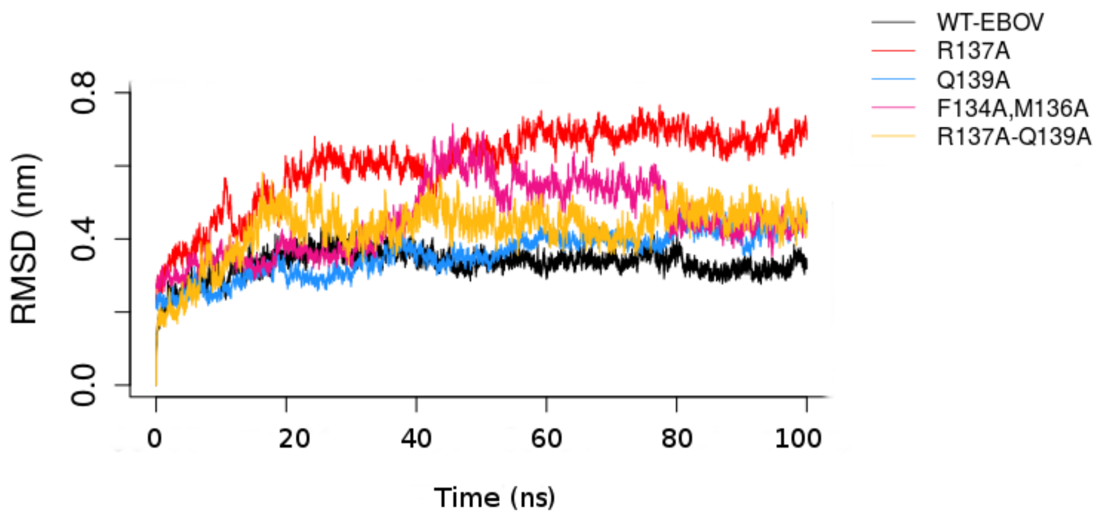

Figure S1: RMSD curves for VP24 KPNA5 complex trajectories. A) Ebola (black line) and Reston (red line) in complex with KPNA5 are shown. The Reston complex showed a higher RMSD that could be explained with the suboptimal binding between the two monomers (red line), while the EBOV complex showed a lower RMSD during the whole 600 ns trajectory, meaning a greater stability of the complex. B) For Ebola VP24 in complex with KPNA5 for wild type and the set of mutated Ebola VP24 proteins.

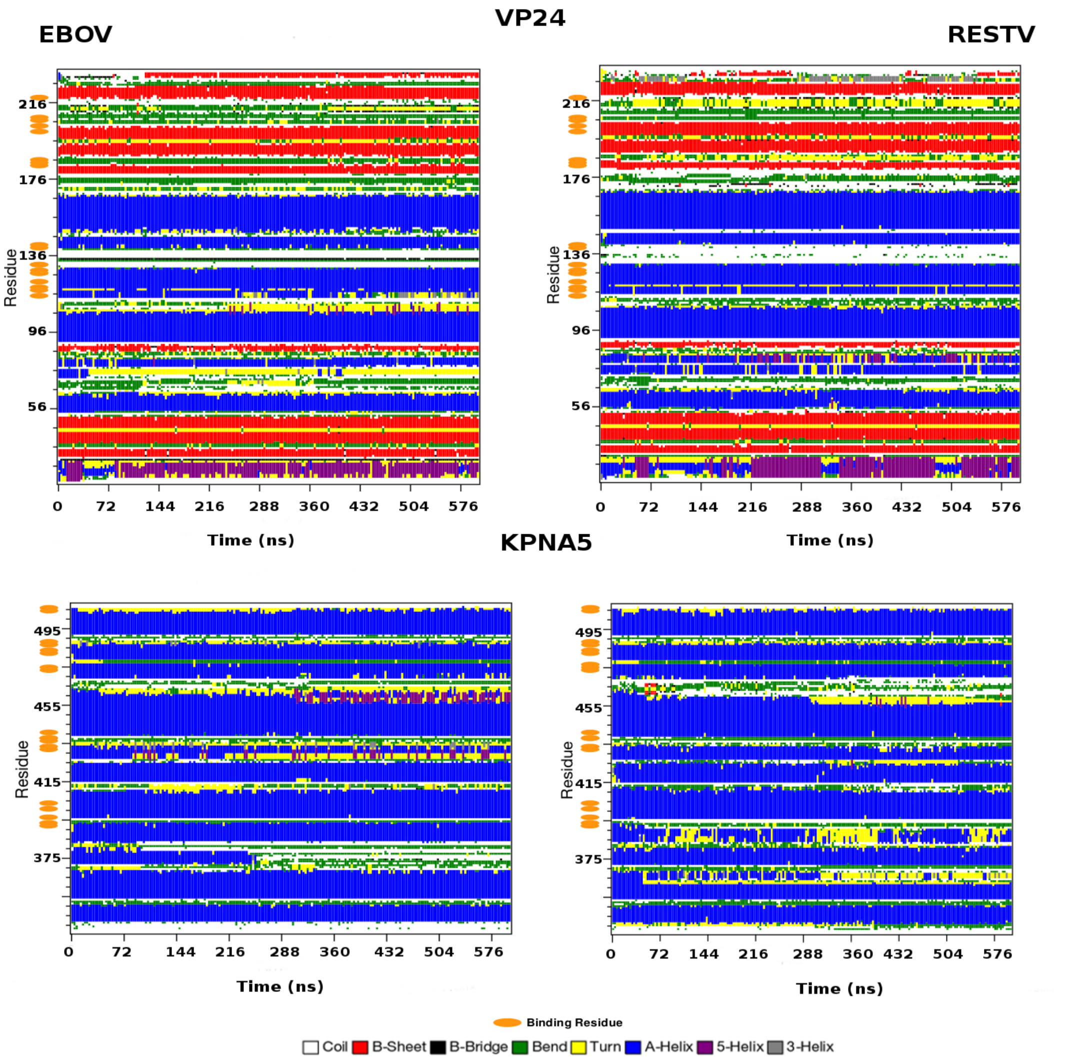

Figure S2: DSSP graphs for the two wild type complexes. On the left side of the graph, the evolution of the EBOV secondary structure is shown in EBOV VP24 (on the top) and KPNA5 (on the bottom). On the right side, the evolution of the RESTV secondary structure is shown for RESTV VP24 (on the top of the graph) and KPNA5 (on the bottom). Residues at the interfaces are mapped in yellow circles.

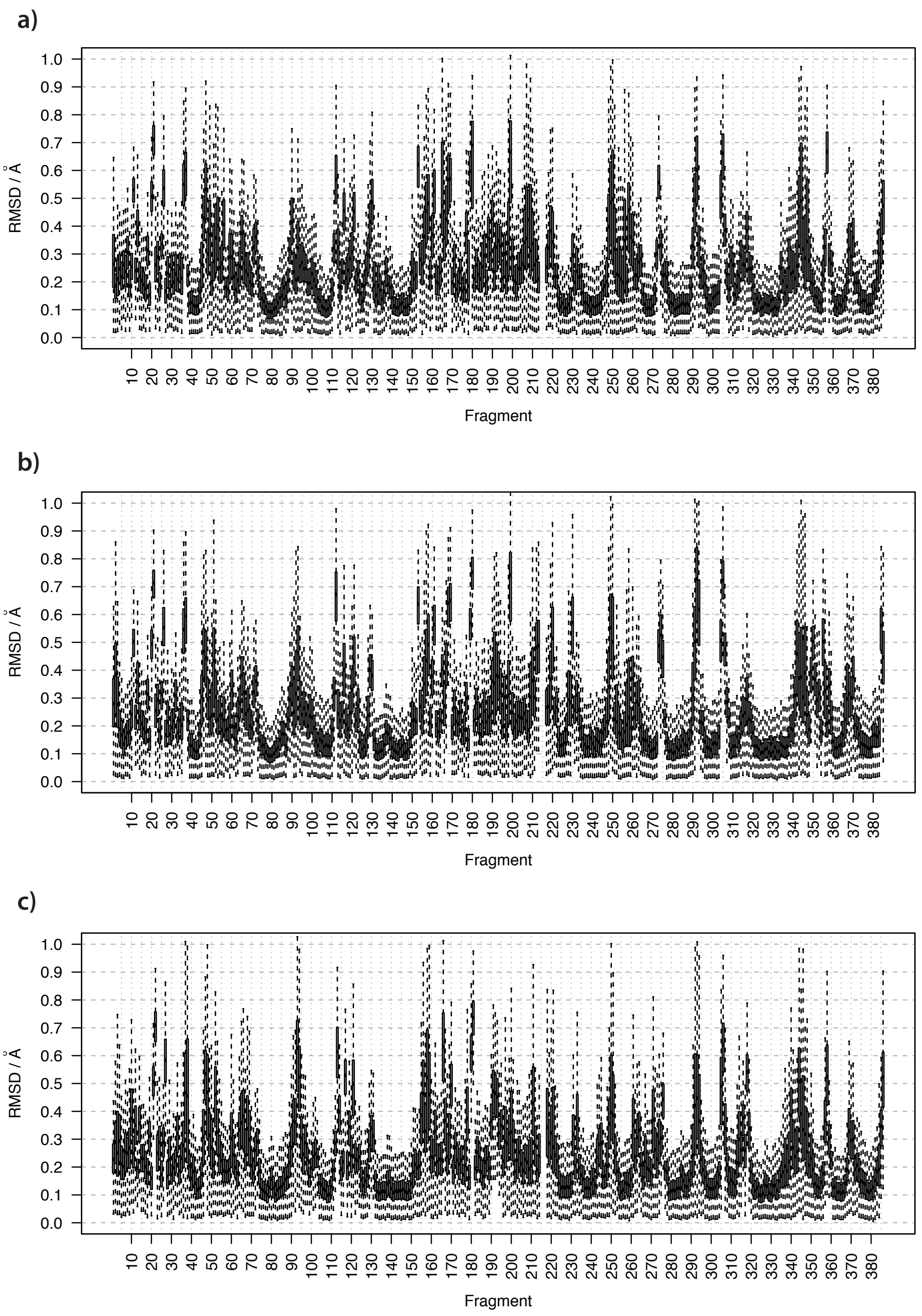

Figure S3: Local error of structural alphabet fit. The RMSD distribution per fragment position was calculated for a) WT EBOV VP24 and KPNA5, b) R137A EBOV VP24 and KPNA5 and c) RESTV VP24 and KPNA5.

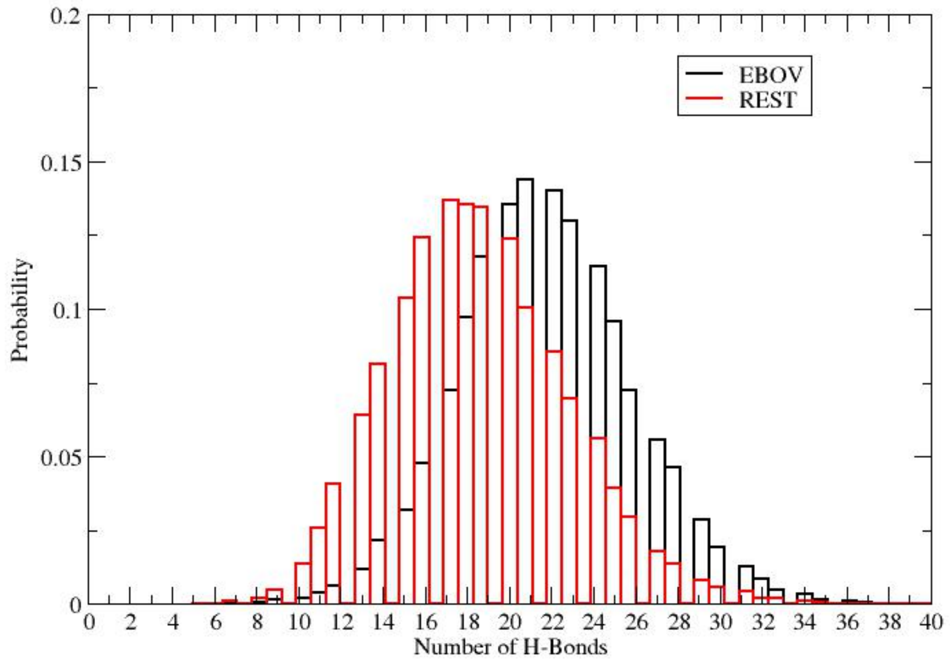

Figure S4: Hydrogen bonding in the Ebola and Reston virus VP24 complexes with KPNA5. Probability distribution of the number of H-bonds at the interface during the simulation for Ebola virus VP24- KPNA5 complex (black) and for Reston virus VP24- KPNA5 (red).
